# Differentially Expressed miRNA-Gene Targets Related to Intramuscular Fat in Musculus Longissimus Dorsi of Charolais × Holstein F_2_-Crossbred Bulls

**DOI:** 10.3390/genes11060700

**Published:** 2020-06-25

**Authors:** Bilal Ahmad Mir, Henry Reyer, Katrin Komolka, Siriluck Ponsuksili, Christa Kühn, Steffen Maak

**Affiliations:** 1 Institute of Muscle Biology and Growth, Leibniz Institute for Farm Animal Biology (FBN), D-18196 Dummerstorf, Germany; toenissen@fbn-dummerstorf.de (K.K.); maak@fbn-dummerstorf.de (S.M.); 2 Institute of Genome Biology, Leibniz Institute for Farm Animal Biology (FBN), D-18196 Dummerstorf, Germany; reyer@fbn-dummerstorf.de (H.R.); ponsuksili@fbn-dummerstorf.de (S.P.); kuehn@fbn-dummerstorf.de (C.K.)

**Keywords:** intramuscular fat, adipogenesis, lipid metabolism, miRNA, microarray, bioinformatics

## Abstract

Intramuscular fat (IMF) is a meat quality indicator associated with taste and juiciness. IMF deposition, influenced by genetic and non-genetic factors, occurs through a transcriptionally coordinated process of adipogenesis. MicroRNAs (miRNAs) are transcriptional regulators of vital biological processes, including lipid metabolism and adipogenesis. However, in bovines, limited data on miRNA profiling and association with divergent intramuscular fat content, regulated exclusively by genetic parameters, have been reported. Here, a microarray experiment was performed to identify and characterize the miRNA expression pattern in the Musculus longissimus dorsi of F_2_-cross (Charolais × German Holstein) bulls with high and low IMF. A total of 38 differentially expressed miRNAs (DE miRNAs), including 33 upregulated and 5 downregulated (corrected *p*-value ≤ 0.05, FC ≥ ±1.2), were reported. Among DE miRNAs, the upregulated miRNAs miR-105a/b, miR-695, miR-1193, miR-1284, miR-1287-5p, miR-3128, miR-3178, miR-3910, miR-4443, miR-4445 and miR-4745, and the downregulated miRNAs miR-877-5p, miR-4487 and miR-4706 were identified as novel fat deposition regulators. DE miRNAs were further analyzed, along with previously identified differentially expressed genes (DEGs) from the same samples and predicted target genes, using multiple bioinformatic approaches, including target prediction tools and co-expression networks, as well as Gene Ontology (GO) and Kyoto Encyclopedia of Genes and Genomes (KEGG) enrichment. We identified DE miRNAs and their gene targets associated with bovine intramuscular adipogenesis, and we provide a basis for further functional investigations.

## 1. Introduction

Intramuscular fat (IMF) content is an important meat quality trait that attributes to taste, tenderness and juiciness [1,2]. Improved beef palatability and quality grade are linked to increased IMF abundance [1]. Deposition of IMF, also known as marbling, is influenced both by genetic (gender, breed and genotype) and non-genetic factors (age, weight, nutrition, castration and stressors) [3,4,5]. Hence, genetic factors could be solely responsible for marbling when non-genetic determinants are identical. Furthermore, marbling is associated with the size and number of adipocytes as well as the fine balance between lipogenesis and the lipolysis rate in muscle [6,7,8]. Adipogenesis (the process of preadipocyte differentiation into mature adipocytes with lipid accumulation) involves hypertrophy and hyperplasia, i.e., the subsequent increase in the size and number of adipocytes [9]. Transcriptional control of adipogenesis is a highly complex and orchestrated process that is mediated by a cascade of expression events of molecules, including coding and non-coding RNA [9,10]. Thus, understanding the molecular regulators of marbling would help in delineating avenues to increase intramuscular fat deposition.

MicroRNAs (miRNAs) are small, endogenous and non-coding RNAs that bind to target mRNA, thereby modulating gene expression by translation inhibition or mRNA breakdown [11,12]. Multiple genes can be regulated by a single miRNA, while a single gene can be modulated by more than one miRNA [13]. MicroRNAs are involved in the fat deposition by regulating the expression of key genes in adipogenesis including *PPARG*, *CEBPα*, *FABP7*, *INSIG1*, *RXR*, *RYR*, *FASN*, *SCD* and *IGF1R*, among others. [14,15,16,17]. Intramuscular adipocyte proliferation and differentiation are influenced by miR-125-5p and miR-23a [15,18]. To date, most of the miRNA functional exploration findings are related to human and murine cell models, and reports of miRNA profiling in bovine fat accretion in general, and IMF in particular, are limited. For instance, profiling of fat tissue in cattle revealed 54 fat-specific miRNAs [19], while 18 miRNAs were concomitant with subcutaneous backfat thickness, with miR-378 showing the highest association [20]. Regarding marbling correlated miRNA profiling, only a few RNA-sequencing studies [21,22,23,24] and a microarray experiment [25] identified divergent miRNAs related to intramuscular fat content. Therefore, profiling of miRNAs in animals with different IMF content is imperative to gain further insight into their potential role in beef fat accretion. Moreover, miRNA regulation of differential IMF deposition in bulls while keeping non-genetic parameters constant has not been reported before.

In a previous study conducted by our group on Musculus longissimus dorsi (MLD) of Charolais × Holstein F_2_-crossbred bulls with low and high IMF content, mRNAs were profiled and differentially expressed genes (DEGs) determined [26]. Here, the same MLD samples were used to identify, through a microarray analysis, differentially expressed miRNAs (DE miRNA) and whether these target and regulate the expression of DEGs. Furthermore, DE miRNA–DEG co-expression network and functional classification was performed, and Gene Ontology (GO) and Kyoto Encyclopedia of Genes and Genomes (KEGG) pathway enrichment elucidation of predicted gene targets were carried out. The bioinformatic analyses revealed miRNA profiles that may be specifically involved in IMF accretion. The variation in IMF of the bulls was exclusively due to genetic differences as non-genetic parameters were kept standardized and identical.

## 2. Materials and Methods

### 2.1. Animals

A total of 20 crossbred bulls from a F_2_-population (Charolais × German Holstein) were assigned to the groups low IMF (1.9 ± 0.6%; *n* = 10) or high IMF (7.0 ± 0.6%; *n* = 10). Three F_1_ sires had an identical number of sons in both groups. The bulls were kept and fed under the standardized condition as described elsewhere [27,28] and slaughtered at an age of 18 months. Samples of Musculus longissimus dorsi (MLD) were shock-frozen in liquid nitrogen and stored until further analyses. The experiment was approved by the Animal Care Committee of the State Mecklenburg-Western Pomerania, Germany (State Office for Agriculture, Food Safety and Fishery; LALLF M-V/TSD/7221.3-2.1-010/03), and husbandry and slaughter conformed to the German Law of Animal Protection.

### 2.2. Samples and RNA Preparation

Small RNAs were isolated from MLD samples of the 20 bulls using a miReasy Mini kit (Qiagen, Hilden, Germany) and subsequently enriched with an RNeasy MinElute Cleanup kit (Qiagen) according to the manufacturer’s instructions. Assessment of the amount and integrity of the isolated small RNAs was done on an Agilent 2100 Bioanalyser (Agilent, Santa Clara, CA, USA) with the Agilent small RNA kit.

### 2.3. MicroRNA Microarray Assay

We used the GeneChip miRNA 3.0 array (Affymetrix, Santa Clara, CA, USA) for miRNA expression profiling essentially as described by the manufacturer. This array included all microRNAs listed in release 17 of miRBase. The microarray is multispecies, covering almost 20,000 miRNAs, including 676 mature miRNAs assigned to *Bos taurus*. Labelling of small RNAs (200 ng/sample) was done with a FlashTag Biotin RNA labelling kit for Affymetrix GeneChip miRNA arrays (Genisphere, Hatfield, PA, USA). Hybridzation of the labelled small RNA with the array lasted 16 h. Subsequent washing and staining were performed in a Fluidics Station 450, and the arrays were then scanned on a G3000 GeneArray Scanner (Affymetrix). Robust multi-array average (RMA) background correction, log2 transformations and quantile normalization methods implemented in JMP Genomics 6 (SAS Institute, Cary, NC, USA) were applied.

### 2.4. Database and Identification of Differentially Expressed miRNAs

The data from the microarray were deposited to Gene Expression Omnibus (GEO) database and are accessible through a GEO Series accession number GSE147622. Here, a similar approach was taken as for the mRNA transcriptome data from the same samples [26] to identify DEGs, but with slight modifications. To identify DE miRNAs, a *t*-test was performed and *p* values were corrected for multiple testing using instructions detailed on the biostathandbook website [29]). MicroRNAs with FC ≥ 1.2 cutoff and a corrected *p*-value of ≤ 0.05 were considered to be statistically different, where FC ≥ 1.2 represented upregulated and FC ≥ −1.2 downregulated miRNAs. Only miRNAs with present calls in at least 7 out of 10 animals per group were selected for further analyses.

### 2.5. Prediction of Potential Target Genes

The miRWalk [30] is an open-source platform that provides predicted and validated miRNA binding sites of human, mouse, rat, dog and cow genes. This database is updated twice a year and provides updated information on miRNA–gene interaction. It uses random-forest-based approach software TarPmiR, searching the complete transcript sequence, including the 5′-UTR, CDS and 3′-UTR [30]. For the determination of mRNA targets of DE miRNAs, 32 previously identified DEGs from the same samples [26] were used. According to established miRNA regulation mechanisms, the upregulated miRNAs were considered to target downregulated DEGs and downregulated miRNAs to target upregulated DEGs. In addition to miRWalk, miRDB [31] and microRNA.org [32] were utilized to strengthen the prediction of targets. Hence, common target genes of functional DE miRNAs from DEGs were determined through these algorithms. However, DE miRNAs can potentially target other genes involved in adipogenesis as well.

### 2.6. Construction of miRNA-Target Co-Expression Interaction Network

The MicroRNA Target Filter tool and core analyses of QIAGEN’s Ingenuity Pathway Analysis (IPA^®^) [33] were used to find co-expression interactions and functional enrichment associations of DE miRNAs and DEGs. The IPA uses TargetScan, miRecords and TarBase as the miRNA target gene database. Given the fact that mammalian miRNAs, as well as their targets sites, are highly evolutionarily conserved, this information is exploited by the IPA^®^ of predicted targets of microRNAs [34,35]. Core analyses and functional enrichment were performed to identify enriched networks and functions associated with fat deposition.

### 2.7. GO Function and KEGG Pathway Enrichment Analysis

Gene Ontology (GO) is a knowledge base used to annotate genes and classify characteristic biological aspects for transcriptome and high-throughput data. Kyoto Encyclopedia of Genes and Genomes (KEGG) is a database that is employed to conduct searches regarding biological pathways, genomes, chemical substances, diseases and drugs. The Database for Annotation, Visualization and Integrated Discovery (DAVID) is an online analysis tool that is utilized to provide a comprehensive functional understanding and biological meaning of large lists of genes [36,37]. List of predicted target genes of DE miRNAs obtained from IPA was filtered by removing those not involved in any pathway or involved in cancer. The remaining target genes in the list were imported to DAVID 6.8. The final target gene list had approximately 60% of DEGs present in it. Key biological processes (BP), cellular components (CC), molecular functions (MF) and KEGG pathways among target genes were analyzed by DAVID.

## 3. Results

### 3.1. Differentially Expressed miRNAs and Their Predicted mRNA Targets

We combined the results of the miRNA array with data on DEGs [26] obtained from the same MLD samples. The analysis was performed based on the criteria mentioned in Section 2.1. A total of 38 DE miRNAs were identified (Corr. *p*-value ≤ 0.05). Among these 38 DE miRNAs, 33 were upregulated (FC ≥ 1.2) and the remaining 5 were downregulated (FC ≥ −1.2) in bulls with high IMF (7%) compared to low IMF (1.9%) content (Table 1). The array was multispecies, hence the miRNAs chosen were from cows, humans and mice because they are highly conserved across mammalian species [34,38]. When predicting mRNA targets, 21 DE miRNAs, including 19 upregulated and 2 downregulated, were identified to interact with predicted DEG targets (Table 2). The predicted mRNA targets were chosen from 32 DEGs identified from a microarray analysis of the same MLD samples [26]. Out of 21 downregulated DEGs, a maximum of 16 were predicted targets of upregulated miRNAs miR-1224 and -1287, while only three targets were predicted for miR-421 (Table 2). However, 8 and 7 upregulated genes were predicted to be targeted by downregulated miRNAs let-7e and miR-877, respectively (Table 2). Other miRNAs were predicted to target 6 to 15 DEGs. The DEGs have been related to IMF deposition previously [26,39], hence DE miRNAs could potentially regulate fat development and accretion by modulating these and other potential gene targets as well as associated pathways.

### 3.2. DE miRNA and DEGs Co-Expression Network and Functional Classification

**Figure 1:**
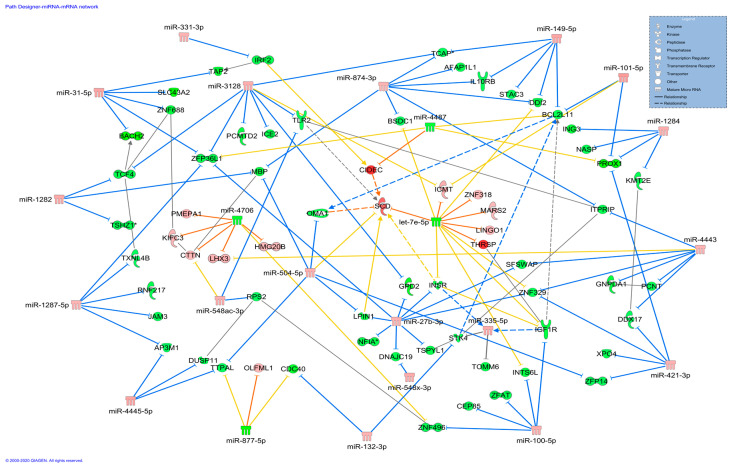
MicroRNA-target gene co-expression network constructed using Ingenuity Pathway Analysis (IPA). Here, upregulation and downregulation of microRNA (miRNA)/gene are represented by red and green, respectively. The intensity of color represents the level of expression, e.g., intense red means higher expression, while light red is low expression of a miRNA or DEG. The gene inhibition and activation by miRNA is shown by blue and orange lines, respectively. Further, predicted targets of the miRNAs are connected with yellow lines. Transcription factors, receptors, mature miRNA etc. are represented by different shapes in a blue box on top right of the image.

**Figure 2:**
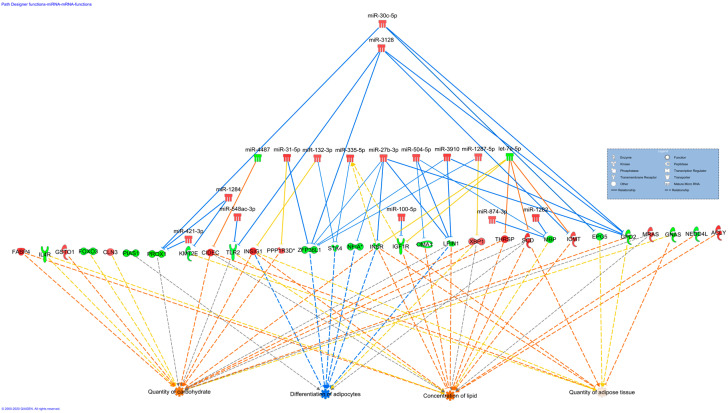
The relationship between differentially expressed miRNAs and their target DEGs with associated biological function related to adipogenesis and fat development. Here, upregulation and downregulation of miRNA/gene are represented by red and green, respectively. The intensity of color represents the level of expression, e.g., intense red means higher expression, while light red is low expression of a miRNA or DEG. The gene inhibition and activation by miRNA is shown by blue and orange lines, respectively. Further, predicted targets of the miRNAs are connected with yellow lines. Transcription factors, receptors, mature miRNA etc. are represented by different shapes in a blue box on top right of the image.

A miRNA–gene co-expression network was constructed to reveal the relationship of DE miRNAs with all DEGs that are basically involved in fat development [26] (Figure 1). The results revealed that, among upregulated miRNAs, miR-27b-3p, miR-31-5p, miR-100-5p, miR-149-5p, miR-421-3p, miR-504, miR-874-3p, miR-1287-5p, miR-1284, miR-4443 and miR-3128 may potentially inhibit the expression of several downregulated DEGs, including *IGF1R*. Contrarily, the downregulated miRNAs let-7e-5p, miR-4706, miR-877-5p and miR-4487 may modulate upregulated DEGs including *THRSP* and *SCD* (Figure 1). Apart from miR-331-3p in upregulated and miR-877-5p in downregulated, the remaining candidates were revealed as nodal miRNAs interacting with two to nine DEGs. The DEGs in the co-expression network are related to lipid metabolism and fat accretion [26]. Similarly, functional enrichment analysis revealed that DE miRNAs regulate DEGs associated with the biological functions “quantity of adipose tissue”, “differentiation of adipocytes”, “concentration of lipid” and “quantity of carbohydrate” (Figure 2).

### 3.3. Gene Ontology (GO) and KEGG Pathway Enrichment Analyses of Target Genes of the DE miRNAs

The predicted gene target list used for Gene Ontology (GO) and KEGG enrichment was obtained from IPA and is available in the supplementary data (Table S1). GO enrichment analysis indicated that DE miRNA gene targets belonged to 1002 biological processes (including cell proliferation regulation, fatty acid metabolic process, regulation of lipid metabolic and cholesterol process), 107 cellular components (including protein-lipid complex, microsome, receptor complex and cell fraction) and 185 molecular functions (including lipid binding, steroid hormone receptor activity, cytokine activity and transcription factor binding) (Table 3). KEGG pathway enrichment analysis revealed that the predicted gene targets of DE miRNAs belonged to 35 pathways, including fatty acid metabolism, Peroxisome proliferator-activated receptor (PPAR) signaling, adipocytokine signaling pathway, glycerolipid and retinol metabolism and type II diabetes mellitus (Table 4). The full list of GO and KEGG enrichment analyses is available in the supplementary data (Table S2).

## 4. Discussion

In our study, we evaluated the differences in miRNA expression in MLD with low and high IMF content, and we identified putative potential target genes from the mRNA microarray from our previous study [26]. The DE miRNAs were related to DEGs and to further gene targets predicted by IPA that may modulate pathways involved in fat development and deposition.

As bulls with varied IMF content were chosen despite the identical husbandry and feeding conditions, in addition to having the same sires, the causal factors for the differences should exclusively be genetic. Hence, differentially expressed genes and miRNAs need to be identified to understand their different regulatory capacity for fat deposition in MLD. Among the DE miRNAs identified in the microarray, some miRNAs are known to have a significant regulatory role in adipogenesis, lipid metabolism and fat tissue development. However, upregulated miRNAs, miR-105a/b, miR-695, miR-1193, miR-1284, miR-1287-5p, miR-3128, miR-3178, miR-3910, miR-4443, miR-4445 and miR-4745, and downregulated miRNAs, miR-877-5p, miR-4487 and miR-4706 are novel to the list of IMF regulators. Further functional investigation and validation of all these miRNAs, especially those interacting with multiple DEGs or involved in the fat-associated biological functions, may reveal whether they play an important role in marbling, lipid and fat biology. This study confirms the expression pattern of various miRNAs that have already been implicated in adipogenesis and fat tissue. For example, increased levels of let-7a in IMF and subcutaneous fat tissue [25,40], miR-101 in serum and adipose tissue of obese individuals [41], miR-1282 in nonalcoholic fat deposition in liver [42], miR-149 in rumen and fat tissue compared to liver, spleen, lungs, kidney and small intestine tissues [43], miR-504 in adipose-derived mesenchymal stem cells of obese individuals [44], miR-132 in the omental fat of obese individuals [45] and miR-421 in fatty hepatic tissues of high fat diet (HFD)-diet mice [46] have been reported. Moreover, miR-10b [47], miR-30c [48,49] miR-132 [50], miR-149 [51], miR-331 [52], miR-335 [53] and miR-425 [54] were all upregulated in adipogenesis and accelerated the process of adipogenic differentiation. Interestingly, miR-10b through targeting *PPARA* [55], miR-101 by modulating phosphatidylserine synthase 1 (*PTDSS1*) and fatty acid elongase 5 (*ELOVL5*) genes [56], miR-100 curtailing *IGF1R* and *SREBF1* [57,58] and miR-135a regulating *IRS2* play important roles in fat accretion, lipid and fatty acid metabolism and insulin signaling. While miR-135a [59] and miR-31 [60,61] are downregulated in 3T3L-1 cellular adipogenesis, they have also been associated with increased glycerol/cholesterol levels [62] and fat deposition through angiotensin [63], respectively. The expression of miR-874 has been confirmed and found increased in cattle with moderate intramuscular fat [24]. To date, concerning the downregulated DE miRNAs, no functional analyses or expression confirmation have been reported. Therefore, further investigation on these miRNAs would reveal their role in and regulation of intramuscular fat deposition.

Target genes of DE miRNAs were enriched for GO terms associated with biological processes, molecular functions and cellular components involved in adipose tissue regulation and lipid metabolism. These findings suggest that these DE miRNAs may play a significant role in the regulation and deposition of fat tissue in bovines. Many of these DE miRNAs, such as miR-27a/b, miR-10b-5p, miR-31-5p, miR-135a and miR-1224-5p, play a range of roles in lipid and fatty acid metabolism, and in fat development by regulating multiple genes [16,17,64,65]. The microRNA-27 family targets *RXRA*, *FASN*, *SREBP1/2*, *PPARA* and *PPARG* to regulate fat and lipid metabolism [14,66,67]. Fat accumulation is promoted by miR-31 inhibition through regulation of *CEBPA* and *PPARG*. MicroRNA-1224 curtails *AMPKA1* to promote hepatic lipogenesis [65] and is also a negative regulator of *TNFA* [68], which may corroborate its potential role in fat development and biology [69]. In this study, predicted targets of miRNAs were also found to be involved in enriched KEGG pathways, including fatty acid metabolism, PPAR signaling, adipocytokine signaling pathway, glycerolipid and retinol metabolism and type II diabetes mellitus. PPAR signaling and downstream targets are essential for differentiation of both in vivo and in vitro differentiation of adipose tissue [70,71]. Moreover, adipocytokine signaling pathway targets have been related to insulin signaling, fat tissue and inflammation [72,73]. Together, GO terms and KEGG pathway enrichment suggest that DE miRNAs target mRNA genes, which are involved in bovine fat development, but this would need to be experimentally verified. Nevertheless, bioinformatic analyses of an informative data set imply the role which DE miRNAs may play in fat accretion and lipid metabolism. Further investigation into DE miRNA expression validation and functional exploration in the fat-deposition-related biological processes is fundamental to elucidate how they regulate IMF development.

## Figures and Tables

**Table 1:** Differentially expressed miRNAs (DE miRNAs) in bulls with high intramuscular fat (IMF) (7.0%) and low IMF (1.9%), corrected *p*-value ≤ 0.05 and the threshold of FC ≥ 1.2.

**Upregulated miRNAs**	**FC**	***p*-Value**	**Corr. *p*-Value**
bta-let-7a-5p	1.36	0.001	0.017
bta-mir-10b-5p	1.35	0.007	0.02
bta-mir-100-5p	1.32	0.003	0.014
bta-mir-101-5p	1.48	0.006	0.02
bta-mir-105a/b	1.39	0.004	0.018
bta-mir-135a-5p	1.31	0.045	0.045
bta-mir-1193	1.33	0.013	0.024
bta-mir-1224-5p	1.26	0.009	0.02
bta-mir-1282	1.38	0.022	0.034
bta-mir-1284	1.31	0.029	0.036
bta-mir-1287-5p	1.20	0.0301	0.036
hsa-mir-27b	1.27	0.023	0.033
hsa-mir-30c-5p	1.24	0.027	0.036
hsa-mir-31-5p	1.36	0.044	0.045
hsa-mir-149-5p	1.34	0.008	0.02
hsa-mir-331-3p	1.21	0.035	0.04
hsa-mir-504-5p	1.3	0.021	0.032
hsa-mir-548a-3p	1.27	0.028	0.036
hsa-mir-874-3p	1.21	0.036	0.04
hsa-mir-3128	1.30	0.005	0.018
hsa-mir-3178	1.21	0.014	0.023
hsa-mir-3910	1.40	0.01	0.02
hsa-mir-4443	1.22	0.008	0.019
hsa-mir-4445-5p	1.47	0.021	0.033
hsa-mir-4745-5p	1.32	0.043	0.045
mmu-mir-30c-5p	1.21	0.029	0.037
mmu-mir-31-5p	1.44	0.004	0.017
mmu-mir-132-3p	1.22	0.032	0.037
mmu-mir-149-5p	1.45	0.008	0.018
mmu-mir-335-5p	1.35	0.008	0.017
mmu-mir-421-3p	1.25	0.024	0.033
mmu-mir-425-5p	1.26	0.042	0.045
mmu-mir-695	1.41	0.002	0.012
**Downregulated miRNAs**	**FC**	***p*-Value**	**Corr. *p*-Value**
bta-let-7e-5p	−1.30	0.0014	0.015
bta-let-7f-5p	−1.52	0.002	0.011
hsa-mir-877-5p	−1.30	0.0139	0.024
hsa-mir-4487	−1.30	0.00004	0.001
hsa-mir-4706	−1.35	0.000006	0.0001

**Table 2:** Predicted DE miRNA gene targets from differentially expressed genes (DEGs) with functions associated with lipid metabolism and fat deposition.

**Upregulated DE miRNA**	**Count**	**Predicted** **Downregulated DEG** **Targets**
miR-1224-5p	16	*ATXN7L1*, *GPD2*, *IL4R*, *PROX1*, *SLC43A2*, *BACH2*, *GNAS*, *RAD50*, *SMARCAD1*, *TAP2*, *CDC40*, *SESN1*, *ART3*, *PPDPFL*, *C2H2orf88*, *TBXAS1*
miR-1287-5p	16	*ATXN7L1*, *GPD2*, *IL4R*, *PROX1*, *SLC43A2*, *BACH2*, *GNAS*, *RAD50*, *SMARCAD1*, *TAP2*, *CDC40*, *ART3*, *PPDPFL*, *C2H2orf88*, *TBXAS1*, *FRAT2*
miR-31-5p	15	*ATXN7L1*, *GPD2*, *IL4R*, *PROX1*, *SLC43A2*, *BACH2*, *GNAS*, *RAD50*, *SMARCAD1*, *TAP2*, *CDC40*, *SESN1*, *ART3*, *PPDPFL*, *TBXAS1*
miR-149-5p	15	*ATXN7L1*, *GPD2*, *IL4R*, *PROX1*, *SLC43A2*, *GNAS*, *RAD50*, *SMARCAD1*, *TAP2*, *CDC40*, *SESN1*, *ART3*, *PPDPFL*, *C2H2orf88*, *FRAT2*
miR-331-3p	15	*ATXN7L1*, *GPD2*, *IL4R*, *PROX1*, *SLC43A2*, *BACH2*, *GNAS*, *RAD50*, *SMARCAD1*, *TAP2*, *SESN1*, *ART3*, *PPDPFL*, *C2H2orf88*, *CETN3*
miR-874-3p	15	*ATXN7L1*, *GPD2*, *IL4R*, *PROX1*, *SLC43A2*, *BACH2*, *GNAS*, *SMARCAD1*, *CDC40*, *SESN1*, *ART3*, *PPDPFL*, *C2H2orf88*, *TBXAS1*, *CETN3*
Let-7b	14	*ATXN7L1*, *GPD2*, *IL4R*, *PROX1*, *SLC43A2*, *BACH2*, *GNAS*, *RAD50*, *SMARCAD1*, *TAP2*, *CDC40*, *SESN1*, *ART3*, *TBXAS1*
miR-425-5p	14	*ATXN7L1*, *GPD2*, *IL4R*, *PROX1*, *SLC43A2*, *BACH2*, *GNAS*, *RAD50*, *SMARCAD1*, *TAP2*, *CDC40*, *SESN1*, *PPDPFL*, *C2H2orf88*
miR-1284	13	*ATXN7L1*, *GPD2*, *IL4R*, *PROX1*, *SLC43A2*, *BACH2*, *RAD50*, *TAP2*, *CDC40*, *SESN1*, *ART3*, *CETN3*, *FRAT2*
miR-1282	12	*ATXN7L1*, *GPD2*, *IL4R*, *PROX1*, *SLC43A2*, *BACH2*, *GNAS*, *RAD50*, *SMARCAD1*, *CDC40*, *SESN1*, *PPDPFL*
miR-504-5p	12	*ATXN7L1*, *GPD2*, *IL4R*, *PROX1*, *SLC43A2*, *BACH2*, *GNAS*, *RAD50*, *SMARCAD1*, *TAP2*, *PPDPFL*, *TBXAS1*
miR-105a	9	*ART3*, *BACH2*, *BACH2*, *CDC40*, *GNAS*, *GPD2*, *GPD2*, *IL4R*, *TBXAS1*
miR-105b	9	*BACH2*, *PPDPFL*, *GNAS*, *GPD2*, *GPD2*, *IL4R*, *PROX1*, *RAD50*, *SLC43A2*
miR-27b-3p	8	*BACH2*, *CETN3*, *IL4R*, *SESN1*, *RAD50*, *SMARCAD1*, *TAP2*, *FRAT2*
miR-132-3p	8	*SLC43A2*, *BACH2*, *CDC40*, *FRAT2*, *GNAS*, *GNAS*, *SESN1*, *SESN1*
miR-100-5p	6	*CDC40*, *GPD2*, *SESN1*, *SLC43A2*, *SMARCAD1*, *CETN3*
miR-10b-5p	6	*ATXN7L1*, *GPD2*, *IL4R*, *RAD50*, *SLC43A2*, *TAP2*
miR-1193	6	*BACH2*, *BACH2*, *GPD2*, *IL4R*, *PROX1*, *TBXAS1*
miR-421-3p	3	*GPD2*, *RAD50*, *SLC43A2*
**Downregulated DE miRNA**	**Count**	**Predicted** **Upregulated DEG** **Targets**
bta-let-7e-5p	8	*ACLY*, *AGRN*, *CIDEC*, *THRSP*, *MRAS*, *SCD*, *INSIG1*, *MGST1*
bta-miR-877-5p	7	*ACLY*, *AGRN*, *CIDEC*, *THRSP*, *MRAS*, *SCD*, *INSIG1*

**Table 3:** Gene Ontology (GO) enrichment analyses of DE miRNA gene targets associated with lipid metabolism and fat accretion.

Category	Term	Count	%	*p*-Value	Fold Enrichment	FDR
GOTERM_BP_FAT	GO:0042127~regulation of cell proliferation	154	12.09741	7.95 × 10^−22^	2.204126802	1.49 × 10^−18^
GOTERM_BP_FAT	GO:0006631~fatty acid metabolic process	43	3.377848	7.30 × 10^−8^	2.446210649	1.37 × 10^−4^
GOTERM_BP_FAT	GO:0019216~regulation of lipid metabolic process	35	2.749411	7.02 × 10^−11^	3.519983347	1.32 × 10^−7^
GOTERM_BP_FAT	GO:0008203~cholesterol metabolic process	29	2.278083	3.13 × 10^−9^	3.550591898	5.86 × 10^−6^
GOTERM_CC_FAT	GO:0000267~cell fraction	179	14.06127	2.80 × 10^−22^	2.06715238	4.09 × 10^−19^
GOTERM_CC_FAT	GO:0005792~microsome	47	3.692066	1.22 × 10^−8^	2.480261257	1.79 × 10^−5^
GOTERM_CC_FAT	GO:0043235~receptor complex	34	2.670856	5.61 × 10^−11^	3.665800661	8.20 × 10^−8^
GOTERM_CC_FAT	GO:0032994~protein-lipid complex	10	0.785546	0.001348	3.573385519	1.952788
GOTERM_MF_FAT	GO:0005125~cytokine activity	55	4.320503	2.43 × 10^−14^	3.129804953	4.04 × 10^−11^
GOTERM_MF_FAT	GO:0003707~steroid hormone receptor activity	21	1.649647	2.33 × 10^−9^	4.755677656	3.87 × 10^−6^
GOTERM_MF_FAT	GO:0008289~lipid binding	69	5.420267	1.34 × 10^−5^	1.701475783	0.022292
GOTERM_MF_FAT	GO:0008134~transcription factor binding	84	6.598586	7.99 × 10^−8^	1.816984056	1.33 × 10^−4^

**Table 4:** Kyoto Encyclopedia of Genes and Genomes (KEGG) pathway enrichment analyses of DE miRNA gene targets associated with lipid and fatty acid metabolism, and fat development.

Category	Term	Count	%	*p*-Value	Fold Enrichment	FDR
KEGG_PATHWAY	hsa04060:Cytokine-cytokine receptor interaction	86	6.755695	1.72 × 10^−11^	2.025634	2.13 × 10^−8^
KEGG_PATHWAY	hsa00562:Inositol phosphate metabolism	23	1.806756	1.31 × 10^−5^	2.628439	0.016234
KEGG_PATHWAY	hsa03320:PPAR signaling pathway	25	1.963865	1.14 × 10^−4^	2.235912	0.141802
KEGG_PATHWAY	hsa00071:Fatty acid metabolism	17	1.335428	2.57 × 10^−4^	2.622725	0.317883
KEGG_PATHWAY	hsa04930:Type II diabetes mellitus	17	1.335428	0.002012	2.232106	2.465567
KEGG_PATHWAY	hsa00561:Glycerolipid metabolism	15	1.178319	0.009253	2.057039	10.88421
KEGG_PATHWAY	hsa00830:Retinol metabolism	17	1.335428	0.00932	1.942759	10.95931
KEGG_PATHWAY	hsa04920:Adipocytokine signaling pathway	17	1.335428	0.06505	1.565806	56.55956

## Supplementary Materials

The following are available online at https://www.mdpi.com/2073-4425/11/6/700/s1. Table S1: DE miRNAs gene targets predicted by IPA; Table S2: GO term and KEGG pathway analyses (complete list).
